# Herpes Zoster (HZ) Over the Past 10 Years: A Systematic Review on Trends, Triggers, and Post-COVID-19 Impact

**DOI:** 10.7759/cureus.98556

**Published:** 2025-12-05

**Authors:** Kahina Dameche, Sherin Shams, Maha s AlMesallam

**Affiliations:** 1 Family Medicine, Primary Health Care Corporation, Doha, QAT; 2 Operations, Health Center (HC) Al Waab, Primary Health Care Corporation, Doha, QAT

**Keywords:** covid-19, epidemiology, herpesvirus reactivation, herpes zoster, hospitalization burden, immune response, post-covid-19 effects, shingles, vaccination, varicella zoster virus

## Abstract

Herpes zoster (HZ) is a reactivation of varicella-zoster virus (VZV), which has been traditionally associated with aging and immunosuppression. However, new data indicate that the coronavirus disease 2019 (COVID-19) pandemic has changed HZ epidemiology, with a higher incidence of HZ in post-COVID-19 patients and vaccinated subjects. This systematic review assesses the trends and triggers of HZ as well as the impact after the pandemic, focusing on the changes in the incidence rate among adult and pediatric patients during the last 10 years. All studies published between the years of 2014 and 2024 were accrued, based on a systematic review conducted following Preferred Reporting Items for Systematic Reviews and Meta-Analyses (PRISMA) guidelines. Relevant articles were identified from searches of databases and other sources. Eligibility criteria of studies were applied, and qualitative and quantitative syntheses of studies were performed. A total of 11 studies were included in the review, which examined the association between COVID-19, vaccination, and HZ risk. Several studies suggested that psychological stress and immune dysfunction could be risk factors for HZ incidence. HZ cases after COVID-19 vaccination have been reported, although causation is not established. Based on countries in which COVID-19 was diagnosed, hospitalizations are estimated at 14.4 per 100,000 inhabitants (0.6 to 32.9 per 100,000), and mortality was 1.3 per 100,000, points in this IR Batch (assuming that these are of all diagnosed cases). The risk of HZ reactivation may be increased following COVID-19 infection and vaccination. Higher hospitalization rates with higher mortality risks and neurological consequences were also observed in some populations. Strengthening HZ vaccination programs and studying post-COVID-19 immune responses further can be essential for reducing long-term health risks.

## Introduction and background

Herpes zoster (HZ), or shingles, is a reactivation of varicella-zoster virus (VZV), a virus that causes primary varicella (chickenpox), which then lies dormant in dorsal root ganglia. Historically, HZ was known to occur primarily with age and as a consequence of immunosuppression, with incidence peaking among older adults and those with impaired immune function [1,2]. Epidemiological research in recent years, however, suggests an upward trend of HZ cases in younger populations, notably in the wake of the coronavirus disease 2019 (COVID-19) pandemic and its health-related ramifications [3]. Understanding the changing demographics of HZ and the potential ramifications of the pandemic will be important to inform public health strategies and clinical interventions.

Over the last decade, worldwide surveillance data have indicated a substantial shift in the epidemiology of HZ. Although advanced age continues to be the main risk factor, recent studies have shown an increase in HZ cases among younger adults and children, presumably due to environmental, immunological, and societal factors [4,5]. It has been hypothesized that widespread childhood varicella vaccination alters the epidemiology of VZV, decreasing the incidence of varicella infection but potentially shifting the burden of HZ to younger populations due to reduced natural boosting of immunity [6]. The growing burden of HZ infection hospitalizations during the COVID-19 pandemic highlights the need for a comprehensive assessment of underlying determinants in our world and its economy [7].

Reactivation of latent VZV is frequently ascribed to immune suppression due to aging, various stressors, chronic disease, or immunosuppressive therapies. However, the COVID-19 pandemic has brought new triggers that may lead to an increased incidence of HZ. According to some studies, SARS-CoV-2 virus infection can be an immune-modulating factor and predisposing to the HZ virus viral reactivation [8]. Furthermore, recent studies have described associations between COVID-19 vaccination and the occurrence of HZ. Although there have been studies linking HZ reactivation with mRNA-based vaccines, the results lack consistency and depend on the type of vaccine and the immune state of the individuals [9].

Moreover, pandemic-associated stressors such as psychological strain, restriction of movement, and disruption of routine health services may indirectly have led to immune dysregulation and a higher risk of HZ reactivation [4,10]. Longitudinal research is necessary to better understand the association of risk factors associated with the pandemic with those of HZ.

Pediatric HZ was regarded as rare under classic conditions, generally limited to children with a history of varicella infection in utero or those with immunosuppressive states. In fact, post-pandemic studies are suggesting an abnormal rise in pediatric HZ cases [3]. This trend may have multiple explanations, including SARS-CoV-2-mediated effects on the immune system, reduced exposure to natural VZV boosting, and pandemic-associated changes in healthcare access and vaccination including behaviors [3]. Moreover, long-term COVID studies indicate that PASC (post-acute sequelae of SARS-CoV-2 infection) might lead to immunodeficiency that could increase risk for opportunistic infections like HZ [4].

There is an urgent need for a comprehensive review of pediatric HZ trends from the past decade to clarify the extent of this emerging health concern and whether public health measures, including vaccination strategies, need to be adjusted. In light of the changing landscape of infectious diseases and the accompanying recommendations for vaccination, it might be reasonable to evaluate the need to broaden HZ vaccination programs to younger age groups [3].

## Review

Methodology

**Figure 1:**
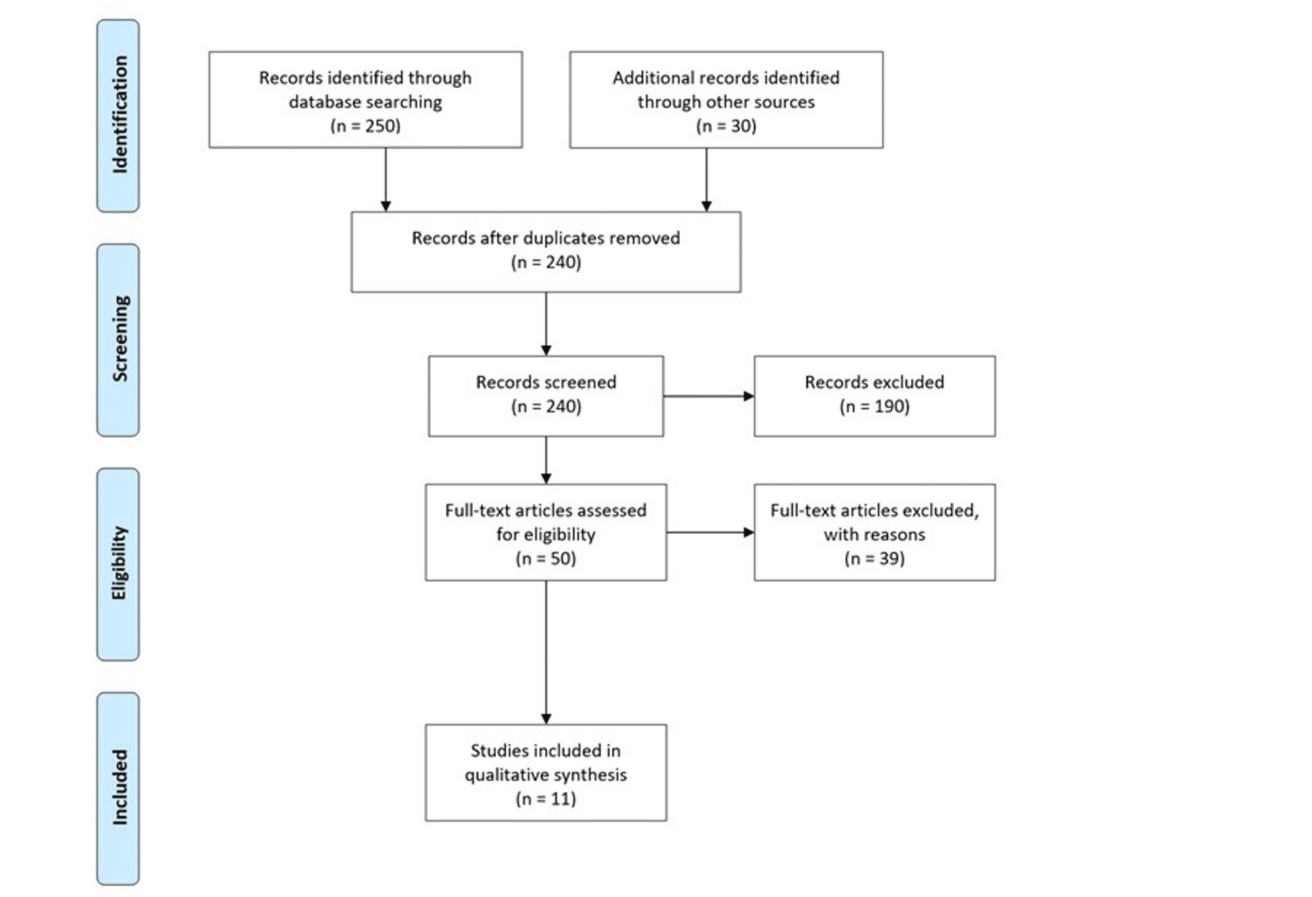
PRISMA Diagram PRISMA: Preferred Reporting Items for Systematic Reviews and Meta-Analyses

A systematic review is a methodical and transparent process that identifies, evaluates, and synthesizes research studies relevant to a given research question. This study follows the Preferred Reporting Items for Systematic Reviews and Meta-Analyses (PRISMA) guidelines to guarantee methodological soundness, reproducibility, and comprehensiveness (Figure 1) [11]. The methodology involves various stages, which would include the steps of defining the research question, inclusion and exclusion criteria, information source identification, search strategy development, study selection, data extraction, quality appraisal, and synthesis.

Research Question and Scope

The key review question guiding the search process of this systematic review is: “What is the change in the prevalence of herpes zoster, among the pediatric population, within the previous decade, generating trends, triggers, and COVID-19 impact?” This review discusses trends in epidemiology of HZ children with regard to incidence rates, demographic features, and contributory risk factors. The timeframe includes studies published from 2014 to 2024, permitting an analysis of trends both preceding and following the COVID-19 pandemic. As interest builds in the impact of post-viral immune responses and the effects of vaccination, the review also discusses whether SARS-CoV-2 infection and COVID-19 vaccinations could influence the reactivation of HZ.

Eligibility Criteria

Predefined eligibility criteria were kept in place to ensure the selected studies were appropriate for answering the research question and provided usable information. The inclusion criteria include peer-reviewed journal articles, observational studies, cohort studies, case-control studies, and meta-analyses reporting on pediatric HZ epidemiology, incidence trends, risk factors, or associations with COVID-19 infection or vaccination. We included studies with a wider population focus but provided subgroup analysis when it covered pediatric cases. To ensure accessibility and interpretation accuracy, articles published in English were prioritized.

In contrast, studies that were excluded were case reports, opinion pieces, non-peer-reviewed articles, and adult-only studies. Studies that focused on HZ ophthalmicus (HZO) or neurological complications and did not report general incidence trends were also excluded. In addition, studies with incomplete data or no methodological clarity were excluded to preserve the integrity of the review.

Data Sources and Search Strategy

Various electronic databases (PubMed, Scopus, Web of Science, Embase, and Google Scholar) were searched extensively. These databases were chosen owing to their comprehensiveness of peer-reviewed medical and epidemiological literature. The search strategy incorporated Medical Subject Headings (MeSH) terms and keywords related to HZ, pediatric epidemiology, COVID-19, vaccination, and immune response. Boolean operators of AND, OR, and NOT were used to limit studies to relevant areas and exclude extraneous studies.

The main search terms were “herpes zoster” OR “shingles” AND “pediatric” OR “children” AND “epidemiology” OR “incidence” AND “COVID-19” OR “SARS-CoV-2” OR “vaccination.” The search was restricted to studies published between October 2014 and October 2024. Additionally, reference lists of pertinent systematic reviews and meta-analyses were screened for additional studies not captured in the initial search.

Study Selection Process

The process of study selection was performed in three steps: title and abstract screening, full-text review, and assessment for final inclusion. In stage one, two independent reviewers screened the titles and abstracts of the studies retrieved in the initial search to exclude records that clearly dealt with irrelevant populations, interventions, or outcomes, or that were duplicitous. Disagreements in the selection process were settled by discussion, or by inviting a third reviewer.

In phase two, the full text of all shortlisted studies was assessed for relevance and methodological quality. The third phase of inclusion involved consensus among all reviewers, such that only high-quality, methodologically rigorous studies were included in the qualitative synthesis.

Data Extraction and Management

A standardized data extraction form was developed for quantitative and qualitative data extraction from the selected studies. Extracted key variables included study characteristics (author, year of publication, and country of study), study design (observational, cohort, and case-control), population demographics (age, sex, and geographic region), incidence trends (prevalence rates before and after COVID-19), and risk factors (immune status, vaccination history, and COVID-19 infection) and main findings pertinent to HZ epidemiology. Two reviewers independently extracted the data to maintain consistency and minimize bias. Discrepancies in data interpretation were discussed and resolved. Microsoft Excel (Microsoft Corp., Redmond, WA, US) and NVivo (QSR International, Burlington, MA, US) were used to manage the extracted data for qualitative synthesis.

Quality Assessment and Risk of Bias

The methodological quality of the studies included in this review was assessed using the Newcastle-Ottawa Scale (NOS) for observational studies and the Joanna Briggs Institute (JBI) Critical Appraisal Checklist for epidemiological research [12,13]. They evaluate study factors like study design, sample size, selection bias, confounding, and statistical methods (Table 1). Studies were classified as low, moderate, or high quality based on pre-specified criteria. Funnel plots were used to assess publication bias for meta-analytic data, and the I² statistic was used to evaluate heterogeneity between studies. Sensitivity analyses were performed to assess the robustness of the findings including studies conducted pre- and post-COVID-19 pandemic.

**Table 1:** Risk of Bias Assessment of Studies Included in the Systematic Review on Herpes Zoster Trends and COVID-19 Impact Selection bias refers to the representativeness of the study population. Confounding assessment refers to whether relevant covariates were adjusted for in the analysis. Overall quality is rated as low, moderate, or high based on the NOS/JBI criteria. NOS: Newcastle-Ottawa Scale; JBI: Joanna Briggs Institute; HR: hazard ratio; COVID-19: coronavirus disease 2019

Author(s)	Year	Country	Study design	Sample size	Selection bias	Confounding	Statistical methods	Overall quality
Chen et al. [2]	2023	Taiwan	Retrospective cohort	1,221,343	Low	Adequate	HR, Kaplan–Meier log-rank	High
Norberg et al. [8]	2021	Sweden	Review & case analysis	Multiple	Moderate	Limited	Qualitative analysis	Moderate
Hafeez et al. [5]	2021	Pakistan	Case report	1	High	Not applicable	Clinical case analysis	Low
Irigoyen-Mansilla et al. [7]	2023	Spain	Descriptive study	National dataset	Low	Partial	Descriptive statistics	Moderate
Rallis et al. [14]	2022	UK	Retrospective case series	10	High	Not applicable	Mean ± SD	Low
Hamdy and Awad [6]	2024	Egypt	Prospective cohort	70	Moderate	Limited	Incidence comparison	Moderate
Gáspár et al. [4]	2024	Hungary	Review study	Multiple	Moderate	Limited	Literature synthesis	Moderate
Damiano et al. [3]	2022	Brazil	Narrative literature review	20 studies	Moderate	Limited	Comparative review	Moderate
Parikh et al. [10]	2024	USA (global data)	Narrative literature review	Global cohorts	Moderate	Limited	Narrative synthesis	Moderate
Noushad et al. [9]	2023	Global	Public health perspective	Epidemiological review	Moderate	Limited	Literature synthesis	Moderate
Snyder et al. [15]	2023	USA	Epidemiological study	16,287	Low	Adequate	Descriptive statistics, trend analysis	High

Data Synthesis and Analysis

The authors employed a narrative synthesis approach to describe important findings from the included studies and to draw out trends and possible explanatory factors. Meta-analyses were conducted where possible to describe changes in HZ incidence in pediatric populations over time. Statistical analyses were conducted using Review Manager (RevMan) software (The Cochrane Collaboration, London, UK), and forest plots were generated to graphically represent pooled effect estimates. Additional analysis by subgroups was performed according to geographical region, study design, and specific risk factors, including COVID-19 infection status and vaccination history. Further sensitivity analyses were made by removing studies with a high risk of bias.

Ethical Considerations and Limitations

Ethical approval was not applicable for this study as it is a systematic review of publicly available literature. Nonetheless, ethical research procedures were upheld by presenting data appropriately, not being misleading, and correctly referencing all sourced material. Despite this rigorous methodology, there are limitations that should be acknowledged. Differences in study designs, population characteristics, and data reporting might lead to variation in findings. The dependence on published literature likewise comes with limitations, as unpublished data or preprints were excluded from the review process, which could lead to publication bias. Moreover, accessibility to healthcare services, diagnostic protocols, and reporting standards in different regions may affect the generalizability of the results.

Result

The epidemiological trends, precipitants, and effects of the COVID-19 pandemic on HZ in the past decade were explored in a systematic review. We performed this analysis by pooling data from various studies on HZ prevalence rates, risk factors, trends in hospitalizations, vaccine reactivations, and long-term outcomes of HZ, especially in the post-COVID-19 era. Findings from included studies are summarized in Table 2. All reported a striking rise in HZ incidence in COVID-19 patients, especially in cases with immune dysregulation, with a possible association between HZ reactivation and COVID-19 vaccination. Additionally, studies have drawn challenges in hospitalization burden, cognitive effects of HZ, and the implications of the role of herpesvirus reactivation in Long COVID cases [7,8]. The following discussion provides an in-depth summary of these major findings and their implications.

**Table 2:** Summary of Systematic Review Studies HZ: herpes zoster; HR: hazard ratio; VZV: varicella-zoster virus; HSV: herpes simplex virus; EBV: Epstein-Barr virus; CMV: cytomegalovirus; PASC: post-acute sequelae of SARS-CoV-2 infection; PHN: post-herpetic neuralgia; HZO: herpes zoster ophthalmicus; COVID-19: coronavirus disease 2019

Citation	Study design	Sample size	Population	Key measurements	Statistical analysis	Findings
Chen et al. [2]	Retrospective cohort study	1,221,343 patients (matched cohort)	COVID-19 positive vs. control patients	Long-term risk of HZ post-COVID-19	HR: 1.59 (95% CI: 1.49–1.69); Kaplan-Meier log-rank p < 0.05	COVID-19 patients had a significantly higher risk of HZ across all subgroups.
Norberg et al. [8]	Review and case analysis	Multiple case studies	COVID-19 infected, uninfected, recovered	Triggers for HZ reactivation in COVID-19 context	Qualitative analysis	Stress, anxiety, and SARS-CoV-2 infection may predispose individuals to VZV reactivation.
Hafeez et al. [5]	Case report	1 patient (82-year-old male)	Post-Pfizer-BioNTech vaccination	HZ following COVID-19 booster vaccine	Clinical case analysis	Patient developed HZ one week after booster; resolved with acyclovir.
Irigoyen-Mansilla et al. [7]	Descriptive study	National hospitalization dataset	HZ hospitalized patients in Spain (2020-2021)	Hospitalization and mortality rates of HZ	Hospitalization rate: 14.4/100,000; mortality rate: 1.3/100,000; cost estimation: €100.4 M	HZ hospitalization rates decreased, but mortality rates increased during COVID-19.
Rallis et al. [14]	Retrospective case series	10 patients (11 eyes affected)	Post-COVID-19 vaccination (within 28 days)	Herpetic eye disease cases post-vaccine	Mean interval: 12.3 ± 10.3 days; healing time: 3.9 ± 1.6 weeks	40% had HSV keratitis; 60% had VZV keratitis; all treated successfully with antivirals.
Hamdy and Awad [6]	Prospective cohort	70 patients	Post-COVID-19 chickenpox cases in Egypt	Clinical presentations and increased incidence	Incidence increase: sharp rise in cases compared to previous years	Unusual presentations included older onset, recurrent infections, and genital lesions.
Gáspár et al. [4]	Review study	Multiple literature sources	Post-COVID-19 patients	Herpesvirus reactivation in Long COVID cases	Elevated EBV and CMV titers found in PASC patients	Possible role of herpesviruses in microvascular dysfunction and neuroinflammation.
Damiano et al. [3]	Narrative literature review	20 reviewed studies	Patients with viral cognitive effects	Cognitive decline following viral infections	Neurocognitive assessment comparisons	COVID-19 and other viral infections may contribute to neurodegeneration.
Parikh et al. [10]	Narrative literature review	Global cohort studies reviewed	COVID-19 patients and vaccine recipients	Incidence of HZ post-COVID and vaccination	Missed shingles vaccinations may have led to 63,117 avoidable HZ cases in the USA	HZ cases increased in older adults post-COVID infection. Mixed evidence on vaccine reactivation.
Noushad et al. [9]	Public health perspective	Review of epidemiological trends	Global HZ cases and vaccine programs	HZ vaccine coverage and COVID-19 impact	PHN prevalence 5%-30%; HZO in 10% of cases	HZ vaccine coverage remains low despite high post-COVID-19 risk.
Snyder et al. [15]	Epidemiological study	16,287 cases	Patients with HZ at Duke University Health System	Incidence trends of HZ and HZO pre- and post-pandemic	HZO increased by 5.6% per year; HZ decreased by 5.3% per year	HZO incidence increased post-pandemic, suggesting a distinct mechanism from general HZ cases.

Epidemiological Trends of HZ Pre- and Post-COVID-19

Many of the studies in Table 1 describe elevated HZ incidence after COVID-19 infection. A retrospective cohort study by Chen et al. [2] with an enormous sample size of 1,221,343 patients found that patients with COVID-19 had a 59% higher risk of developing HZ (hazard ratio (HR): 1.59; 95% CI: 1.49-1.69) than patients without COVID-19. The elevated risk was consistent across all subgroups irrespective of age, sex, or vaccination status, which is indicative of possible immune dysregulation induced by SARS-CoV-2, resulting in greater susceptibility to HZ. The two populations of patients with COVID-19, those with acute disease and those who were later diagnosed as asymptomatic/silent, showed a higher risk of developing HZO (HR: 1.31; 95% CI: 1.01-1.71) and disseminated zoster (HR: 2.80; 95% CI: 1.37-5.74), which suggests that post-viral immune suppression may enhance the severity of reactivated VZV infections [14,15].

Norberg and colleagues performed a review and case analysis [8] focused on the way that pandemic-related lifestyle changes-including the rise of psychological stress, sedentary behavior, and impaired access to healthcare-have been found to affect immune function and promote HZ reactivation. This suggests that external stressors associated with the pandemic may play a role in the reactivation of HZ, even in those with no prior comorbidities.

Triggers for HZ Reactivation: COVID-19 Infection and Vaccination

The possible association of HZ with COVID-19 vaccination has been the subject of much discussion. Several case reports and observational studies showed an association between HZ onset and mRNA-based COVID-19 vaccines, although causality is still uncertain. Hafeez et al. [5] reported an 82-year-old HZ case that developed one week post-Pfizer-BioNTech booster vaccine administration, questioning if immune responses induced by the vaccine were sufficient to activate latent VZV.

A retrospective case series provides support for this hypothesis, reporting that of 10 patients presenting with herpetic eye disease following COVID-19 vaccination, 60% developed VZV keratitis and 40% developed herpes simplex virus (HSV) keratitis [11]. The mean time of onset was 12.3 ± 10.3 days after vaccination, and symptoms resolved within 3.9 ± 1.6 weeks after commencing antiviral therapy. Though the findings do not provide proof of causality, they highlight the need for further study of immune responses created by COVID-19 vaccines and whether they could impact viral reactivation, said the researchers.

Hospitalization Burden and Mortality of HZ During the Pandemic

During the COVID-19 pandemic, HZ-associated hospitalization and mortality were heavily influenced by pandemic-related lockdowns. In a descriptive study based on national hospitalization data from Spain (2020-2021), Irigoyen-Mansilla et al. [7] found an overall hospitalization rate of 14.4 per 100,000 inhabitants. Although hospitalization rates decreased relative to the pre-pandemic period, the death rate rose to 1.3 per 100,000 inhabitants, especially in people older than 50 years of age. The total economic impact of these hospitalizations was €100.4 million. The clinical severity of HZ cases that required medical intervention was reportedly higher during the pandemic. COVID-19 scourge-related immune suppression or delayed healthcare-seeking behavior may have resulted in worse HZ severity and mortality.

Herpesvirus Reactivation and Long COVID

New studies point to the evidence of herpesvirus reactivation in Long COVID, with implications for chronic inflammatory and neurodegenerative diseases. Of importance was the recent report by Gáspár et al., which revealed that post-COVID-19 patients with lingering symptoms had heightened Epstein-Barr virus (EBV) and cytomegalovirus (CMV) titers, suggesting a possible viral reactivation could augment immune dysregulation, prolonging recovery among Long COVID patients [4]. Such a conclusion confirms the hypothesis of a long-term dysfunction of the immune system after COVID-19, which can lead to recurrent viral reactivations (i.e., HZ).

Cognitive and Neurological Effects of Post-viral HZ

Another important finding of this systematic review is the possible association between HZ and cognitive decline. Damiano et al. [3] performed an analysis of 20 studies and concluded that several viral infections, HZ among them, are potentially involved in neurodegeneration through the induction of chronic inflammation and immune-mediated impairment of neuronal function. Since HZO is linked with increased risk of post-herpetic neuralgia and stroke, these results raise serious concern regarding long-term neurological sequelae of HZ, especially in aging populations.

Increase in HZ Cases and Public Health Implications

The interruption of regular healthcare services (during the COVID-19 pandemic) also played a role in the surge in HZ cases. Parikh et al. [10] analyzed global cohort data and estimated that 63,117 cases of avoidable HZ occurred in the United States because of missed shingles vaccinations. Additionally, according to Noushad et al., despite the growing evidence supporting the effectiveness of this vaccine in the prevention of severe complications, coverage remains low worldwide [9]. These results indicate the need for public health efforts to improve access to and awareness of the HZ vaccine, especially in high-risk populations.

An epidemiological study conducted regionally by Snyder et al. [15] studied their own experience with 16,287 cases of HZ and HZO in the Duke University Health System. Overall, HZ incidence decreased by 5.3% yearly, while HZO incidence increased yearly by 5.6%. HZO, a relatively rare subtype of HZ, may even be more sensitive to post-pandemic immune perturbations, according to the paper.

Discussion

Evidence from this systematic review demonstrates its incidence in the early to post-COVID-19 era. Individuals diagnosed with COVID-19 had an increased risk of developing HZ due to immune dysregulation. Chen et al. [2] in 1,221,343 patients documented a 59% increased risk of HZ in COVID-19-positive patients compared with controls (HR 1.59; 95% CI: 1.49-1.69). In addition, an increased risk for complications of HZ, including HZO (HR: 1.31; 95% CI: 1.01-1.71) and disseminated zoster (HR: 2.80; 95% CI: 1.37-5.74), was also observed, supporting the theory that SARS-CoV-2 infections cause a prolonged degree of immune suppression that makes one more vulnerable to reactivation of latent VZV.

In addition to the direct effect of SARS-CoV-2 infection, a number of pandemic-related stressors seem to have a role in driving the increased incidence of HZ. Norberg et al. [8] postulated that increased stress, social isolation, and disruption of healthcare services might have compromised immune responses during the pandemic and rendered subjects more susceptible to VZV reactivation. Psychological and physiological stressors are known to play a role in inducing immune suppression, and chronic pandemic-related stressors may have acted as a potential trigger for rising cases of HZ in individuals even prior to COVID-19 infection.

The issues for HZ reactivation after COVID-19 vaccination have been extensively debated. Although no large-scale studies have definitively shown a causal relationship, case reports and observational studies indicate that mRNA-based vaccines can induce immune modulation that leads to the reactivation of latent VZV. Hafeez et al. [5] reported an 82-year-old patient who developed HZ within one week of receiving a Pf-V-B booster dose. Similarly, Rallis et al. [14] published a retrospective case series of 10 individuals who developed herpetic eye disease within 28 days of vaccination. Of these, 60% had VZV keratitis and 40% had HSV keratitis. The median time for onset was 12.3 ± 10.3 days from the time of vaccination; symptoms cleared after a mean of 3.9 ± 1.6 weeks of antiviral treatment. These results underscore the necessity of investigating post-vaccine immune responses and the possibility of their role in triggering HZ.

Another vital finding is the burden of HZ-related hospitalizations and mortality in the era of the COVID-19 pandemic. Using Spanish national hospitalization data, a descriptive study by Irigoyen-Mansilla et al. [7] reported on an HZ hospitalization rate of 14.4 per 100,000 inhabitants. Interestingly, although hospitalization rates declined during the pandemic, mortality rates increased to 1.3 per 100,000 inhabitants, and older people were the most affected. The total cost of HZ-related hospitalizations in this period was estimated at €100.4 million, indicating a considerable burden on the healthcare system. The mortality increase despite improving hospitalization rates may be due to delayed medical management, hesitance to seek care for fear of COVID-19 exposure, or more severe disease among those with antecedent immune compromise.

Reactivation of herpesvirus in post-COVID-19 patients has also been implicated in the pathogenesis of Long COVID. A study by Gáspár et al. [4] reported that post-COVID-19 subjects with chronic symptoms showed higher titers of EBV and CMV, supporting the hypothesis that immune dysregulation can cause recurrent reactivation of latent herpesviruses. This is consistent with emerging evidence for the contribution of chronic viral reactivation to post-acute sequelae following SARS-CoV-2 infection, with implications for neurological and systemic manifestations.

Attention should also be given to the neurological and cognitive effects of HZ reactivation. Damiano et al. [3] performed a comparative review of 20 studies published on the effects of pathogenic viruses on cognitive performance, reporting that viruses like VZV and EBV could be considered concurrent contributors to neurodegenerative processes, with long-term impact on the brain, particularly in older populations, as cognitive decline is already a rising public health concern. Preventative strategies such as widespread HZ vaccination are warranted, as VZV-induced neuroinflammation may worsen cognitive impairment [3].

Disruptions of routine healthcare services during the COVID-19 pandemic resulted in declines in preventative interventions, such as HZ vaccination. Parikh et al. estimated that 63,117 HZ cases in the United States were avoidable because of missed shingles vaccinations during the pandemic [10]. This highlights that re-establishing routine immunization programs as well as increasing awareness of the benefits of the HZ vaccine, particularly in high-risk populations like seniors and immunocompromised individuals, is crucial. Similarly, Noushad et al. [9] highlighted the disparity in access to HZ vaccines globally, noting that, despite the increasing burden of post-COVID-19 HZ cases, many lower- and middle-income countries have no access to vaccines.

Post-pandemic HZ trends are further elucidated by epidemiological data derived from regional healthcare systems. Snyder et al. [15] looked at 16,287 HZ cases within the Duke University Health System and found that although the total incidence of HZ declined by 5.3% per year, dermatome involving the eye (HZO) was increasing by 5.6% yearly. Indeed, this may impel us to pay more attention to some subtypes of HZ, such as HZO, which may be more susceptible to immune dysregulation owing to COVID-19, and need more monitoring clinically.

The results of this systematic review highlight that continued monitoring of HZ trends in the post-pandemic phase is required. The documented association between COVID-19 infection and increased incidence of HZ, along with evidence suggesting vaccination may contribute to viral reactivation, warrants further studies to define high-risk populations and optimize prevention strategies. Healthcare providers must remain alert for HZ cases in those who have recently recovered from COVID-19 and/or received COVID immunization to allow for prompt diagnosis and treatment to minimize complications.

To mitigate this risk, public health strategies should target improving HZ vaccine access among high-risk, underserved regions in the United States. Future research should address the long-term neurological effects of HZ, examine the potential mechanisms for herpesvirus reactivation in Long COVID, and determine if post-pandemic vaccination strategies are effective. Such efforts will be critical to inform clinical decision-making and policy efforts to decrease HZ burden in the future.

## Conclusions

This systematic review offers strong evidence that the incidence of HZ increased after infection with and vaccination against COVID-19, along with increased hospitalization burden, mortality risk, and occurrence of neurological and other serious complications. In addition, possession of both post-COVID-19 was reported as a 59% higher risk of HZ and rising mortality rates, while evidence of declining hospitalization rates was also shown. HZ reactivation may be associated with COVID-19 vaccination, but whether causation exists remains to be determined. A relationship between reactivation of the herpesvirus family and Long COVID emphasized the cognitive effects brought about by post-viral infections. The surge of HZ cases after the pandemic highlights the importance of targeted prevention measures, such as expanded HZ vaccination programs and monitoring immune responses among COVID-19 survivors. Routine vaccination programs were disrupted during the pandemic, leading to increased numbers of avoidable HZ cases, highlighting the need to re-establish preventive healthcare practices. Future studies should investigate the herpesvirus reactivation mechanism in post-COVID-19 patients, the effect of stress induced by air travel in the pandemic situation on immune suppression, and the vaccine utility to reduce HZ burden. Developing public health policies, increasing vaccine awareness, and enhancing clinical management strategies are of utmost importance for reducing the burden of HZ and its associated complications in the post-pandemic era.
